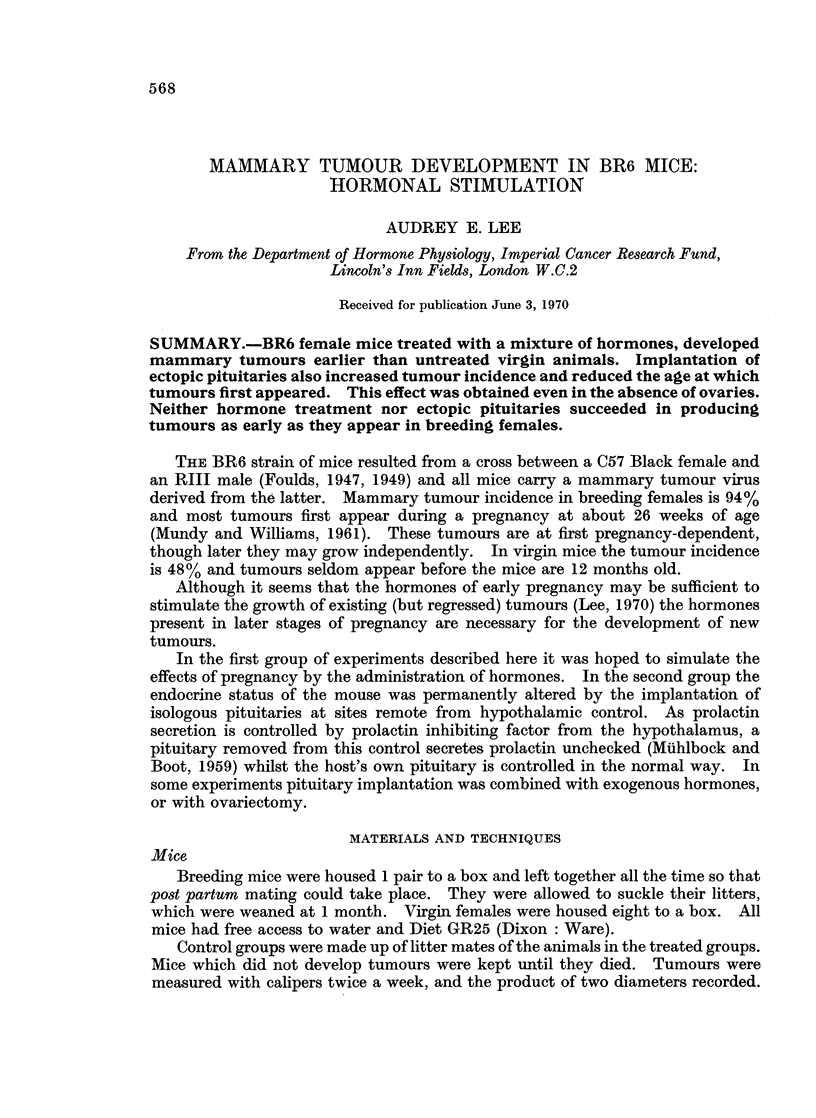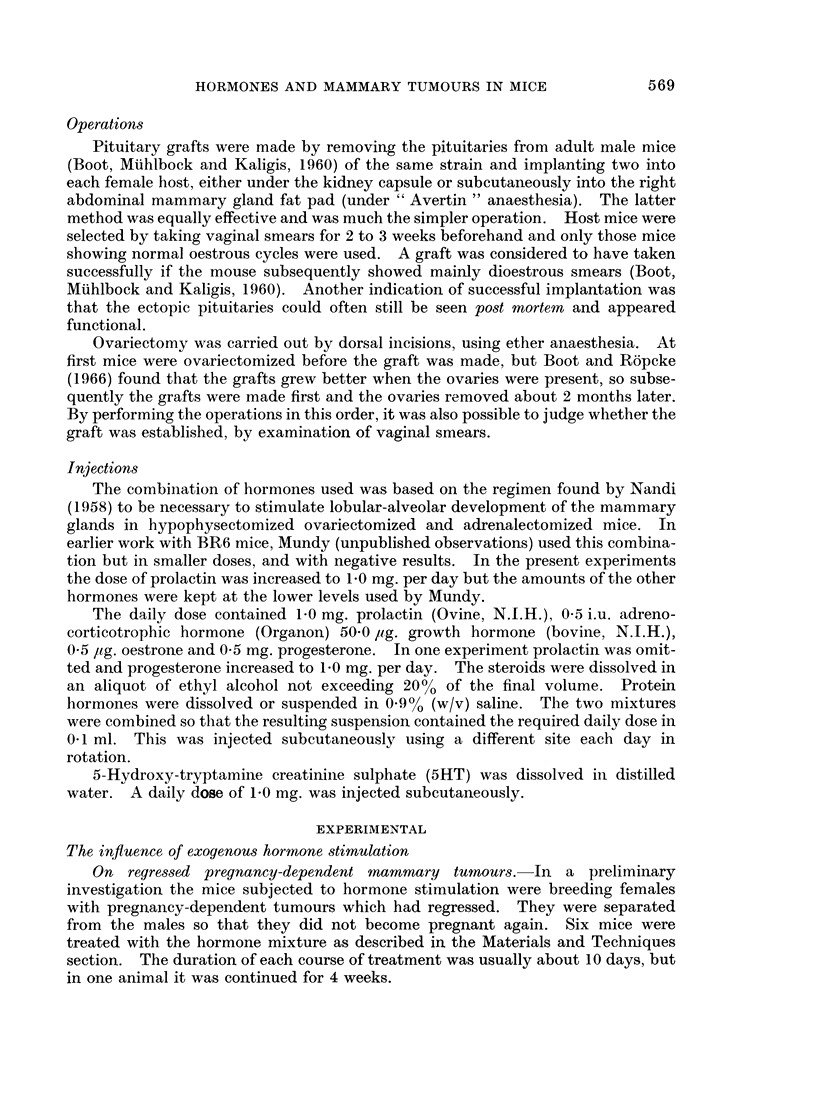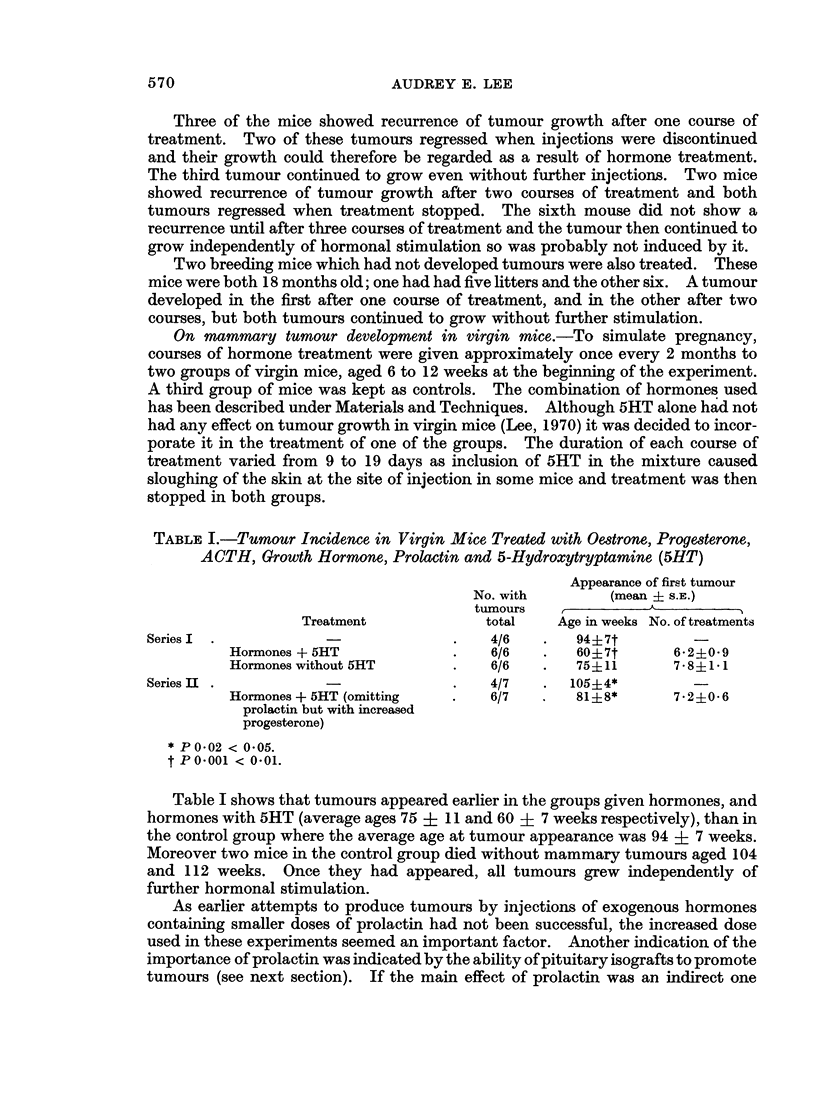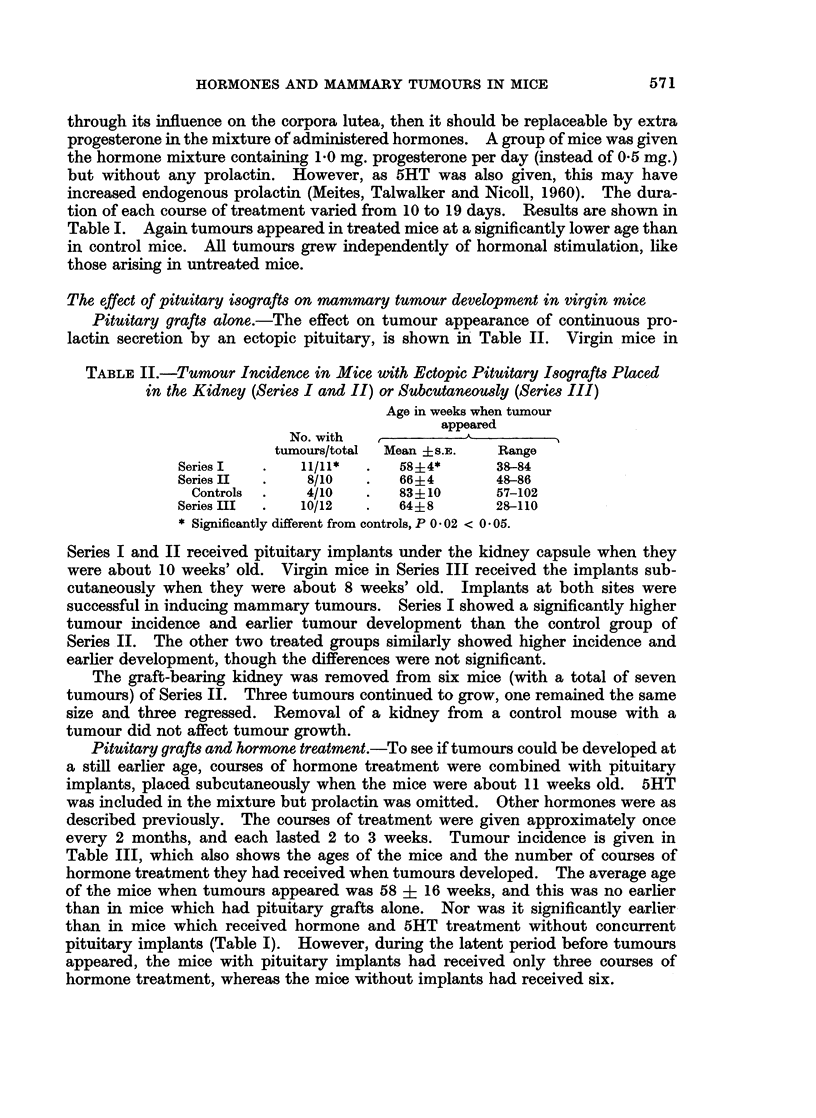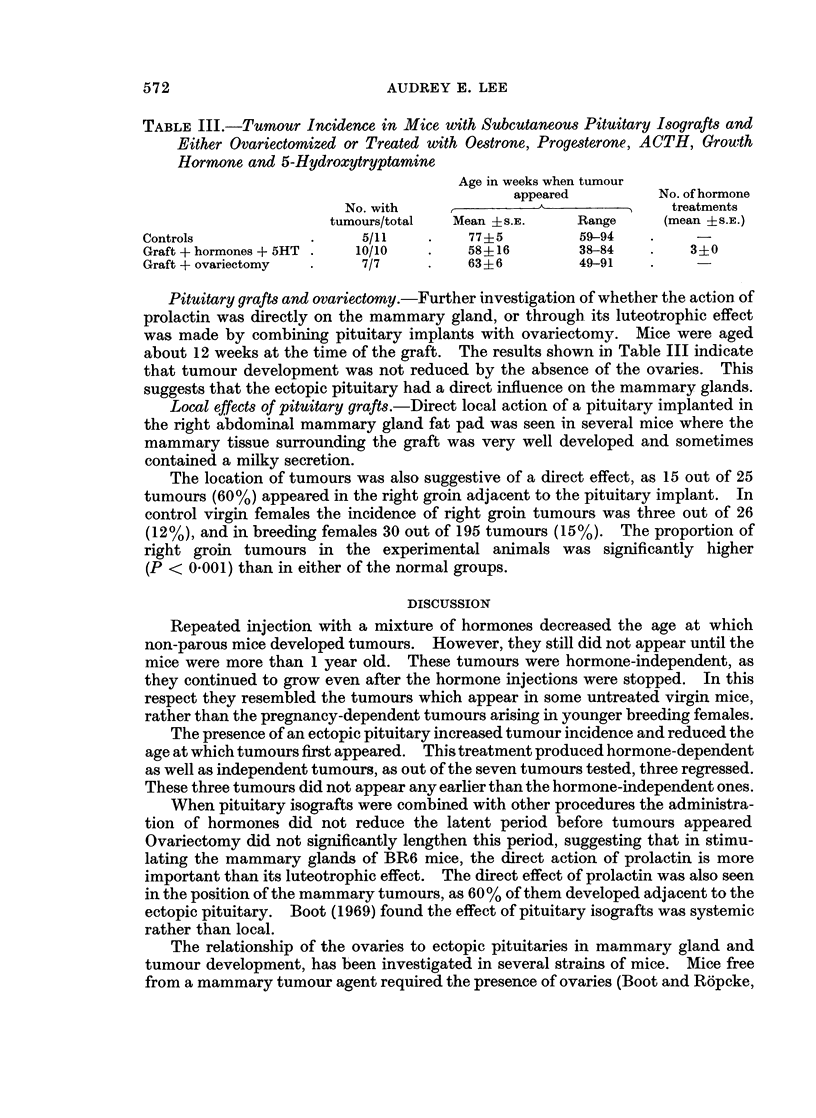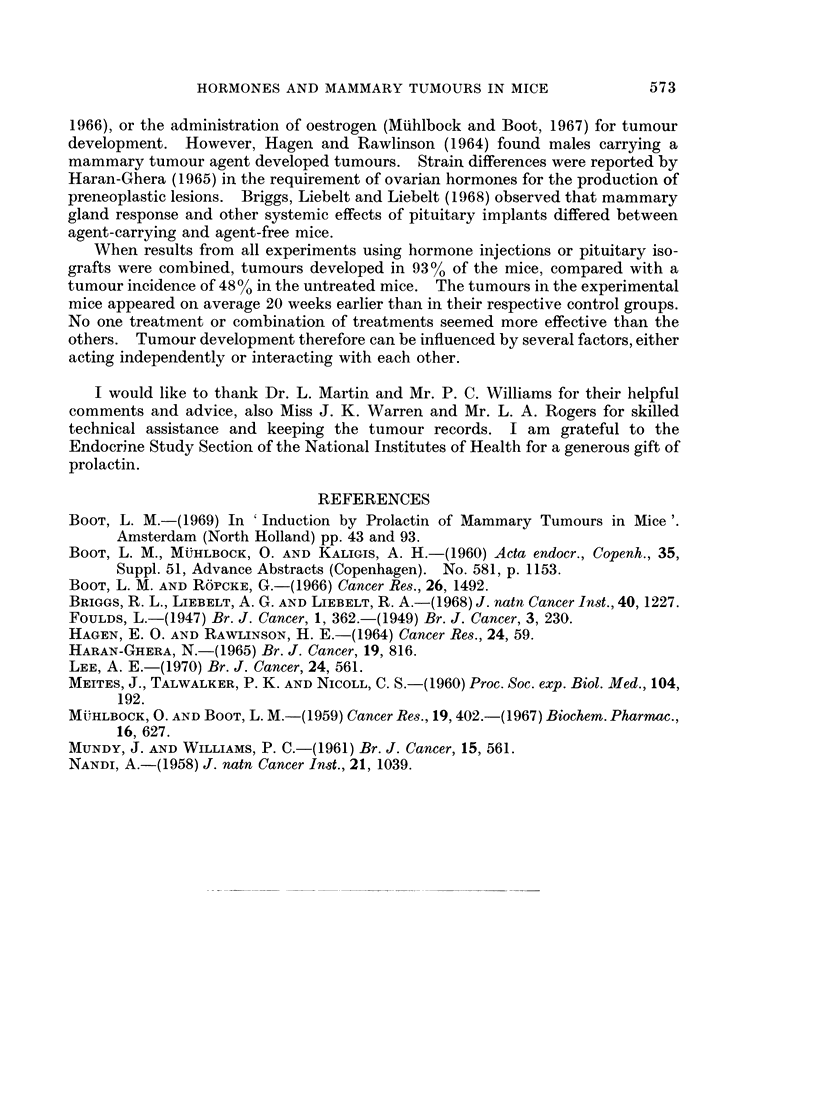# Mammary Tumour Development in BR6 Mice: Hormonal Stimulation

**DOI:** 10.1038/bjc.1970.69

**Published:** 1970-09

**Authors:** Audrey E. Lee

## Abstract

BR6 female mice treated with a mixture of hormones, developed mammary tumours earlier than untreated virgin animals. Implantation of ectopic pituitaries also increased tumour incidence and reduced the age at which tumours first appeared. This effect was obtained even in the absence of ovaries. Neither hormone treatment nor ectopic pituitaries succeeded in producing tumours as early as they appear in breeding females.


### MAMMARY TUMOUR DEVELOPMENT IN BR6 MICE: HORMONAL STIMULATION

AUDREY E. LEE

*From the Department of Hormone Physiology, Imperial Cancer Research Fund, Lincoln's Inn Fields, London W.C.2*

Received for publication June 3, 1970

**SUMMARY.—BR6 female mice treated with a mixture of hormones, developed mammary tumours earlier than untreated virgin animals. Implantation of ectopic pituitaries also increased tumour incidence and reduced the age at which tumours first appeared. This effect was obtained even in the absence of ovaries. Neither hormone treatment nor ectopic pituitaries succeeded in producing tumours as early as they appear in breeding females.**

THE BR6 strain of mice resulted from a cross between a C57 Black female and an RIII male (Foulds, 1947, 1949) and all mice carry a mammary tumour virus derived from the latter. Mammary tumour incidence in breeding females is 94% and most tumours first appear during a pregnancy at about 26 weeks of age (Mundy and Williams, 1961). These tumours are at first pregnancy-dependent, though later they may grow independently. In virgin mice the tumour incidence is 48% and tumours seldom appear before the mice are 12 months old.

Although it seems that the hormones of early pregnancy may be sufficient to stimulate the growth of existing (but regressed) tumours (Lee, 1970) the hormones present in later stages of pregnancy are necessary for the development of new tumours.

In the first group of experiments described here it was hoped to simulate the effects of pregnancy by the administration of hormones. In the second group the endocrine status of the mouse was permanently altered by the implantation of isologous pituitaries at sites remote from hypothalamic control. As prolactin secretion is controlled by prolactin inhibiting factor from the hypothalamus, a pituitary removed from this control secretes prolactin unchecked (Mühlbock and Boot, 1959) whilst the host's own pituitary is controlled in the normal way. In some experiments pituitary implantation was combined with exogenous hormones, or with ovariectomy.

### MATERIALS AND TECHNIQUES

### *Mice*

Breeding mice were housed 1 pair to a box and left together all the time so that *post partum* mating could take place. They were allowed to suckle their litters, which were weaned at 1 month. Virgin females were housed eight to a box. All mice had free access to water and Diet GR25 (Dixon : Ware).

Control groups were made up of litter mates of the animals in the treated groups. Mice which did not develop tumours were kept until they died. Tumours were measured with calipers twice a week, and the product of two diameters recorded.

*Operations*

Pituitary grafts were made by removing the pituitaries from adult male mice (Boot, Mühlbock and Kaligis, 1960) of the same strain and implanting two into each female host, either under the kidney capsule or subcutaneously into the right abdominal mammary gland fat pad (under " Avertin " anaesthesia). The latter method was equally effective and was much the simpler operation. Host mice were selected by taking vaginal smears for 2 to 3 weeks beforehand and only those mice showing normal oestrous cycles were used. A graft was considered to have taken successfully if the mouse subsequently showed mainly dioestrous smears (Boot, Mühlbock and Kaligis, 1960). Another indication of successful implantation was that the ectopic pituitaries could often still be seen *post mortem* and appeared functional.

Ovariectomy was carried out by dorsal incisions, using ether anaesthesia. At first mice were ovariectomized before the graft was made, but Boot and Röpcke (1966) found that the grafts grew better when the ovaries were present, so subsequently the grafts were made first and the ovaries removed about 2 months later. By performing the operations in this order, it was also possible to judge whether the graft was established, by examination of vaginal smears.

*Injections*

The combination of hormones used was based on the regimen found by Nandi (1958) to be necessary to stimulate lobular-alveolar development of the mammary glands in hypophysectomized ovariectomized and adrenalectomized mice. In earlier work with BR6 mice, Mundy (unpublished observations) used this combination but in smaller doses, and with negative results. In the present experiments the dose of prolactin was increased to 1·0 mg. per day but the amounts of the other hormones were kept at the lower levels used by Mundy.

The daily dose contained 1·0 mg. prolactin (Ovine, N.I.H.), 0·5 i.u. adrenocorticotrophic hormone (Organon) 50·0 $\mu$g. growth hormone (bovine, N.I.H.), 0·5 $\mu$g. oestrone and 0·5 mg. progesterone. In one experiment prolactin was omitted and progesterone increased to 1·0 mg. per day. The steroids were dissolved in an aliquot of ethyl alcohol not exceeding 20% of the final volume. Protein hormones were dissolved or suspended in 0·9% (w/v) saline. The two mixtures were combined so that the resulting suspension contained the required daily dose in 0·1 ml. This was injected subcutaneously using a different site each day in rotation.

5-Hydroxy-tryptamine creatinine sulphate (5HT) was dissolved in distilled water. A daily dose of 1·0 mg. was injected subcutaneously.

### EXPERIMENTAL

*The influence of exogenous hormone stimulation*

*On regressed pregnancy-dependent mammary tumours.*—In a preliminary investigation the mice subjected to hormone stimulation were breeding females with pregnancy-dependent tumours which had regressed. They were separated from the males so that they did not become pregnant again. Six mice were treated with the hormone mixture as described in the Materials and Techniques section. The duration of each course of treatment was usually about 10 days, but in one animal it was continued for 4 weeks.

Three of the mice showed recurrence of tumour growth after one course of treatment. Two of these tumours regressed when injections were discontinued and their growth could therefore be regarded as a result of hormone treatment. The third tumour continued to grow even without further injections. Two mice showed recurrence of tumour growth after two courses of treatment and both tumours regressed when treatment stopped. The sixth mouse did not show a recurrence until after three courses of treatment and the tumour then continued to grow independently of hormonal stimulation so was probably not induced by it.

Two breeding mice which had not developed tumours were also treated. These mice were both 18 months old; one had had five litters and the other six. A tumour developed in the first after one course of treatment, and in the other after two courses, but both tumours continued to grow without further stimulation.

*On mammary tumour development in virgin mice.*—To simulate pregnancy, courses of hormone treatment were given approximately once every 2 months to two groups of virgin mice, aged 6 to 12 weeks at the beginning of the experiment. A third group of mice was kept as controls. The combination of hormones used has been described under Materials and Techniques. Although 5HT alone had not had any effect on tumour growth in virgin mice (Lee, 1970) it was decided to incorporate it in the treatment of one of the groups. The duration of each course of treatment varied from 9 to 19 days as inclusion of 5HT in the mixture caused sloughing of the skin at the site of injection in some mice and treatment was then stopped in both groups.

TABLE I.—*Tumour Incidence in Virgin Mice Treated with Oestrone, Progesterone, ACTH, Growth Hormone, Prolactin and 5-Hydroxytryptamine (5HT)*

| | Treatment | No. with tumours total | Appearance of first tumour (mean ± S.E.) | |
|---|---|---|---|---|
| | | | Age in weeks | No. of treatments |
| Series I | — | 4/6 | 94±7† | — |
| | Hormones + 5HT | 6/6 | 60±7† | 6·2±0·9 |
| | Hormones without 5HT | 6/6 | 75±11 | 7·8±1·1 |
| Series II | — | 4/7 | 105±4* | — |
| | Hormones + 5HT (omitting prolactin but with increased progesterone) | 6/7 | 81±8* | 7·2±0·6 |

* *P* 0·02 < 0·05.

† *P* 0·001 < 0·01.

Table I shows that tumours appeared earlier in the groups given hormones, and hormones with 5HT (average ages 75 ± 11 and 60 ± 7 weeks respectively), than in the control group where the average age at tumour appearance was 94 ± 7 weeks. Moreover two mice in the control group died without mammary tumours aged 104 and 112 weeks. Once they had appeared, all tumours grew independently of further hormonal stimulation.

As earlier attempts to produce tumours by injections of exogenous hormones containing smaller doses of prolactin had not been successful, the increased dose used in these experiments seemed an important factor. Another indication of the importance of prolactin was indicated by the ability of pituitary isografts to promote tumours (see next section). If the main effect of prolactin was an indirect one through its influence on the corpora lutea, then it should be replaceable by extra progesterone in the mixture of administered hormones. A group of mice was given the hormone mixture containing 1·0 mg. progesterone per day (instead of 0·5 mg.) but without any prolactin. However, as 5HT was also given, this may have increased endogenous prolactin (Meites, Talwalker and Nicoll, 1960). The duration of each course of treatment varied from 10 to 19 days. Results are shown in Table I. Again tumours appeared in treated mice at a significantly lower age than in control mice. All tumours grew independently of hormonal stimulation, like those arising in untreated mice.

*The effect of pituitary isografts on mammary tumour development in virgin mice*

*Pituitary grafts alone.*—The effect on tumour appearance of continuous prolactin secretion by an ectopic pituitary, is shown in Table II. Virgin mice in

TABLE II.—*Tumour Incidence in Mice with Ectopic Pituitary Isografts Placed in the Kidney (Series I and II) or Subcutaneously (Series III)*

| | No. with tumours/total | Age in weeks when tumour appeared: Mean ±S.E. | Range |
|---|---|---|---|
| Series I | 11/11* | 58±4* | 38–84 |
| Series II | 8/10 | 66±4 | 48–86 |
| Controls | 4/10 | 83±10 | 57–102 |
| Series III | 10/12 | 64±8 | 28–110 |

* Significantly different from controls, $P$ 0·02 < 0·05.

Series I and II received pituitary implants under the kidney capsule when they were about 10 weeks' old. Virgin mice in Series III received the implants subcutaneously when they were about 8 weeks' old. Implants at both sites were successful in inducing mammary tumours. Series I showed a significantly higher tumour incidence and earlier tumour development than the control group of Series II. The other two treated groups similarly showed higher incidence and earlier development, though the differences were not significant.

The graft-bearing kidney was removed from six mice (with a total of seven tumours) of Series II. Three tumours continued to grow, one remained the same size and three regressed. Removal of a kidney from a control mouse with a tumour did not affect tumour growth.

*Pituitary grafts and hormone treatment.*—To see if tumours could be developed at a still earlier age, courses of hormone treatment were combined with pituitary implants, placed subcutaneously when the mice were about 11 weeks old. 5HT was included in the mixture but prolactin was omitted. Other hormones were as described previously. The courses of treatment were given approximately once every 2 months, and each lasted 2 to 3 weeks. Tumour incidence is given in Table III, which also shows the ages of the mice and the number of courses of hormone treatment they had received when tumours developed. The average age of the mice when tumours appeared was 58 ± 16 weeks, and this was no earlier than in mice which had pituitary grafts alone. Nor was it significantly earlier than in mice which received hormone and 5HT treatment without concurrent pituitary implants (Table I). However, during the latent period before tumours appeared, the mice with pituitary implants had received only three courses of hormone treatment, whereas the mice without implants had received six.

TABLE III.—*Tumour Incidence in Mice with Subcutaneous Pituitary Isografts and Either Ovariectomized or Treated with Oestrone, Progesterone, ACTH, Growth Hormone and 5-Hydroxytryptamine*

| | No. with tumours/total | Age in weeks when tumour appeared: Mean ±S.E. | Range | No. of hormone treatments (mean ±S.E.) |
|---|---|---|---|---|
| Controls | 5/11 | 77±5 | 59–94 | — |
| Graft + hormones + 5HT | 10/10 | 58±16 | 38–84 | 3±0 |
| Graft + ovariectomy | 7/7 | 63±6 | 49–91 | — |

*Pituitary grafts and ovariectomy.*—Further investigation of whether the action of prolactin was directly on the mammary gland, or through its luteotrophic effect was made by combining pituitary implants with ovariectomy. Mice were aged about 12 weeks at the time of the graft. The results shown in Table III indicate that tumour development was not reduced by the absence of the ovaries. This suggests that the ectopic pituitary had a direct influence on the mammary glands.

*Local effects of pituitary grafts.*—Direct local action of a pituitary implanted in the right abdominal mammary gland fat pad was seen in several mice where the mammary tissue surrounding the graft was very well developed and sometimes contained a milky secretion.

The location of tumours was also suggestive of a direct effect, as 15 out of 25 tumours (60%) appeared in the right groin adjacent to the pituitary implant. In control virgin females the incidence of right groin tumours was three out of 26 (12%), and in breeding females 30 out of 195 tumours (15%). The proportion of right groin tumours in the experimental animals was significantly higher ($P < 0{\cdot}001$) than in either of the normal groups.

### DISCUSSION

Repeated injection with a mixture of hormones decreased the age at which non-parous mice developed tumours. However, they still did not appear until the mice were more than 1 year old. These tumours were hormone-independent, as they continued to grow even after the hormone injections were stopped. In this respect they resembled the tumours which appear in some untreated virgin mice, rather than the pregnancy-dependent tumours arising in younger breeding females.

The presence of an ectopic pituitary increased tumour incidence and reduced the age at which tumours first appeared. This treatment produced hormone-dependent as well as independent tumours, as out of the seven tumours tested, three regressed. These three tumours did not appear any earlier than the hormone-independent ones.

When pituitary isografts were combined with other procedures the administration of hormones did not reduce the latent period before tumours appeared Ovariectomy did not significantly lengthen this period, suggesting that in stimulating the mammary glands of BR6 mice, the direct action of prolactin is more important than its luteotrophic effect. The direct effect of prolactin was also seen in the position of the mammary tumours, as 60% of them developed adjacent to the ectopic pituitary. Boot (1969) found the effect of pituitary isografts was systemic rather than local.

The relationship of the ovaries to ectopic pituitaries in mammary gland and tumour development, has been investigated in several strains of mice. Mice free from a mammary tumour agent required the presence of ovaries (Boot and Röpcke,

1966), or the administration of oestrogen (Mühlbock and Boot, 1967) for tumour development. However, Hagen and Rawlinson (1964) found males carrying a mammary tumour agent developed tumours. Strain differences were reported by Haran-Ghera (1965) in the requirement of ovarian hormones for the production of preneoplastic lesions. Briggs, Liebelt and Liebelt (1968) observed that mammary gland response and other systemic effects of pituitary implants differed between agent-carrying and agent-free mice.

When results from all experiments using hormone injections or pituitary isografts were combined, tumours developed in 93% of the mice, compared with a tumour incidence of 48% in the untreated mice. The tumours in the experimental mice appeared on average 20 weeks earlier than in their respective control groups. No one treatment or combination of treatments seemed more effective than the others. Tumour development therefore can be influenced by several factors, either acting independently or interacting with each other.

I would like to thank Dr. L. Martin and Mr. P. C. Williams for their helpful comments and advice, also Miss J. K. Warren and Mr. L. A. Rogers for skilled technical assistance and keeping the tumour records. I am grateful to the Endocrine Study Section of the National Institutes of Health for a generous gift of prolactin.

### REFERENCES

Boot, L. M.—(1969) In ' Induction by Prolactin of Mammary Tumours in Mice '. Amsterdam (North Holland) pp. 43 and 93.

Boot, L. M., Mühlbock, O. and Kaligis, A. H.—(1960) *Acta endocr., Copenh.*, **35**, Suppl. 51, Advance Abstracts (Copenhagen). No. 581, p. 1153.

Boot, L. M. and Röpcke, G.—(1966) *Cancer Res.*, **26**, 1492.

Briggs, R. L., Liebelt, A. G. and Liebelt, R. A.—(1968) *J. natn Cancer Inst.*, **40**, 1227.

Foulds, L.—(1947) *Br. J. Cancer*, **1**, 362.—(1949) *Br. J. Cancer*, **3**, 230.

Hagen, E. O. and Rawlinson, H. E.—(1964) *Cancer Res.*, **24**, 59.

Haran-Ghera, N.—(1965) *Br. J. Cancer*, **19**, 816.

Lee, A. E.—(1970) *Br. J. Cancer*, **24**, 561.

Meites, J., Talwalker, P. K. and Nicoll, C. S.—(1960) *Proc. Soc. exp. Biol. Med.*, **104**, 192.

Mühlbock, O. and Boot, L. M.—(1959) *Cancer Res.*, **19**, 402.—(1967) *Biochem. Pharmac.*, **16**, 627.

Mundy, J. and Williams, P. C.—(1961) *Br. J. Cancer*, **15**, 561.

Nandi, A.—(1958) *J. natn Cancer Inst.*, **21**, 1039.